# Development and validation of machine learning-driven prediction model for serious bacterial infection among febrile children in emergency departments

**DOI:** 10.1371/journal.pone.0265500

**Published:** 2022-03-25

**Authors:** Bongjin Lee, Hyun Jung Chung, Hyun Mi Kang, Do Kyun Kim, Young Ho Kwak

**Affiliations:** 1 Department of Pediatrics, Seoul National University Hospital, Seoul, Korea; 2 Department of Emergency Medicine, CHA Bundang Medical Center, CHA University, Seongnam, Korea; 3 Department of Pediatrics, College of Medicine, The Catholic University of Korea, Seoul, Korea; 4 Department of Emergency Medicine, Seoul National University Hospital, Seoul, Korea; Sreenidhi Institute of Science and Technology, INDIA

## Abstract

Serious bacterial infection (SBI) in children, such as bacterial meningitis or sepsis, is an important condition that can lead to fatal outcomes. Therefore, since it is very important to accurately diagnose SBI, SBI prediction tools such as ‘Refined Lab-score’ or ‘clinical prediction rule’ have been developed and used. However, these tools can predict SBI only when there are values of all factors used in the tool, and if even one of them is missing, the tools become useless. Therefore, the purpose of this study was to develop and validate a machine learning-driven model to predict SBIs among febrile children, even with missing values. This was a multicenter retrospective observational study including febrile children <6 years of age who visited Emergency departments (EDs) of 3 different tertiary hospitals from 2016 to 2018. The SBI prediction model was trained with a derivation cohort (data from two hospitals) and externally tested with a validation cohort (data from a third hospital). A total of 11,973 and 2,858 patient records were included in the derivation and validation cohorts, respectively. In the derivation cohort, the area under the receiver operating characteristic curve (AUROC) of the RF model was 0.964 (95% confidence interval [CI], 0.943–0.986), and the area under the precision-recall curve (AUPRC) was 0.753 (95% CI, 0.681–0.824). The conventional LR (CLR) model showed corresponding values of 0.902 (95% CI, 0.894–0.910) and 0.573 (95% CI, 0.560–0.586), respectively. In the validation cohort, the AUROC (95% CI) of the RF model was 0.950 (95% CI, 0.945–0.956), the AUPRC was 0.605 (95% CI, 0.593–0.616), and the CLR presented corresponding values of 0.815 (95% CI, 0.789–0.841) and 0.586 (95% CI, 0.553–0.619), respectively. We developed a machine learning-driven prediction model for SBI among febrile children, which works robustly despite missing values. And it showed superior performance compared to CLR in both internal validation and external validation.

## Introduction

Fever is one of the most common reasons that children visit the emergency department (ED) [1]. In the post-pneumococcal conjugate vaccine (PCV) era, the incidences of serious bacterial infections (SBI) have significantly decreased, and the most common cause of fever in children that visit the ED is self-limiting viral infections [2]. However, determination of the etiology of fever is nevertheless an important task especially as SBIs in children, such as bacterial meningitis or sepsis, are still primarily encountered at the ED. If the diagnosis of SBI is missed or delayed, it can lead to serious complications and even death. In infants under 3 months of age, fever may be the only indicator of SBI. Accordingly, several studies have been conducted to find predictors of SBI in febrile children.

Each clinical aspect from febrile children can be used to estimate the probability of SBI [3], from the peak or duration of fever, capillary refill time [4], well-known biochemical markers such as C reactive protein (CRP) and procalcitonin (PCT) [5], to some novel biomarkers that have been evaluated as candidates for predicting SBI [6]. Furthermore, ideas combining each of the parameters to improve the predictive performances have been examined. In a multicenter cohort study of children under 3 years old, the ‘Refined Lab-score’ was suggested as a predictor that used PCT, CRP, and dipstick urinalysis [7]. In another study involving infants less than 60 days old, the ‘clinical prediction rule’ was also introduced using the absolute neutrophil count (ANC), urinalysis, and PCT [8]. These studies have shown favorable predictive power. However, because the aforementioned score or rule depends on completed and reported tests results, predictions cannot be made under the presence of missing values, which is a limitation. Therefore, in resource-limited circumstances or patients without specific test results, these methods are not applicable.

Recently, with the remarkable development of information technology, studies in various fields—such as risk prediction and diagnosis—are being actively conducted and incorporated into medicine [9–12]. In addition, with machine learning algorithms, various methods of processing ‘missing values’ have been introduced, which make it easier to cope with missing values more flexibly than traditional methods [13–16]. On the other hand, missing values may have been measured but omitted from data collection or may have not been measured because the clinician may have determined it unnecessary at initial evaluation. If so, it would be necessary to use it as an important predictor of clinical judgment rather than being excluded from the predictive model or imputation due to the omission from the data collection process.

In this study, we aimed to develop a model to predict SBI among patients who visited the pediatric ED for fever using a machine learning methodology to reflect the clinical meaning of missing values. Furthermore, the machine learning prediction model developed was compared with a prediction model developed by traditional logistic regression (LR), and an external and internal validation was performed.

## Materials and methods

### Study design and setting

This retrospective observational study was conducted at three university-affiliated hospitals (Seoul National University [SNU] Hospital, SNU Bundang Hospital, and Seoul Metropolitan Government [SMG]—SNU Boramae Medical Center). From August 2016 to February 2018, patients under 6 years of age with fever who visited the pediatric EDs of the above hospitals were registered in ‘The SNU Fever Registry’, which was used to conduct this study. This registry included demographic information such as age and sex, clinical information such as fever onset and accompanying symptoms, and information such as which laboratory tests were performed and corresponding test results.

### Data preprocessing and definitions

Among the records in the registry, suspected keystroke errors (that is, values that are generally difficult to consider physiological) were excluded in analyses (e.g., heart rate over 300 beats per minute or respiratory rate over 120 breaths per minute). The data were divided into categorical and continuous variables for preprocessing. Continuous variables were divided into two groups: age-dependent and age-independent. Age-dependent variables (variables whose normal range varies depending on age, such as heart rate and respiratory rate) were analyzed by calculating z-scores according to age using the ‘generalized additive models for location, scale and shape’ package and the ‘sitar’ package of R software [17, 18]. Continuous variables were feature scaled through standardization, and missing values among continuous variables were imputed as the mean value of the corresponding variable values. Categorical variables were converted through one-hot encoding for machine learning. Missing values of categorical variables were not imputed, and the missing value itself was used for machine learning as a new variable through one-hot encoding.

SBI was defined as laboratory-proven bacteremia, urinary tract infection (UTI), lobar pneumonia, bacterial central nervous system (CNS) infection, and septic arthritis or osteomyelitis as defined in a previous study [7]. Laboratory-proven bacteremia was defined as the identification of bacteria in blood culture, and UTI was also defined when more than 5 × 10^4^ colonies/mL were cultured in catheterized or mid-stream catch urine specimens. Lobar pneumonia was defined based on chest radiogram readings by board-certified radiologists. Bacterial CNS infection was defined as positive cerebrospinal fluid culture, and septic arthritis or osteomyelitis was defined as positive blood or joint fluid culture(s).

### Prediction model development and validation

Among the three hospitals’ data, data from the two hospitals (SNU Hospital and SNU Bundang Hospital) were classified as the derivation cohort, and the data from the other hospital (SMG-SNU Boramae Medical Center) were classified as the validation cohort.

In the case of analyzing formal registry data, previous studies reported that the difference in performance between machine learning algorithms was not significant [12, 19]. Therefore, we decided to select a machine learning algorithm to find the difference from the conventional method, rather than paying attention to the comparison of machine leaning algorithms. We selected random forest (RF) as the machine learning algorithm, because this study used somewhat formalized data from the registry. In-depth algorithms such as deep learning would not be necessary. In addition, the fact that RF could also show the importance of each feature used for classification using Gini impurity influenced the selection. By calculating the information gain of each feature through the difference in GINI impurity when dividing the decision tree, how much each feature contributes to the prediction was shown, and the ‘feature importance’ function of the python scikit-learn library was used in this process [12, 20, 21].

The prediction model was derived using the five-fold cross-validation method using the data of the derivation cohort, and internal validation was performed. The five-fold cross-validation method divides the data into 5 splits, learning in 4 of them, testing in the remaining 1, and performs the test split 5 times without overlapping. This method was used to minimize the distortion of the results that can occur by dividing the training set and the test set by specific splits. External validation was performed by applying each of these 5 models to the validation cohort.

In addition to the prediction model using machine learning, a model to predict SBI using an LR analysis method, which is traditionally used in prediction model development, was used to compare the predictive performance. This analysis method was defined as conventional LR (CLR) because it used a typical existing method, and variables used in RF were also used in CLR. After performing univariable LR analysis for each variable, statistically significant variables with a *P* value < 0.05 were used to develop a multivariable analysis model. The final multivariable LR model was derived through a backward selection process. Similar to the RF model, the CLR model was derived using the data of the derivation cohort, internally validated, and externally validated using the validation cohort data.

R version 4.0.1 (R Foundation for Statistical Computing, Vienna, Austria) was used for data preprocessing and conventional multivariate LR analysis. Python and open libraries such as scikit-learn were used to develop the machine learning model [20].

### Outcome measures

The primary outcome of this study was the performance of prediction models in the validation cohort, and the secondary outcome was the predictive performance in the derivation cohort. The area under the receiver operating characteristic curve (AUROC) and area under the precision-recall curve (AUPRC) were used to evaluate the predictive performance.

‘Accuracy’ can show skewed results when evaluating the performance of models trained on imbalanced datasets; thus, indicators such as ‘precision’ (positive predictive value) and ‘recall’ (sensitivity) are more commonly used, and these are often collectively expressed as the AUPRC. Since the dataset of this study was expected to be imbalanced (the number of SBI cases and non-SBI cases were not the same), the AUPRC together with the AUROC were used to evaluate the predictive performance. Like AUROC, the higher the AUPRC values are, the better the performance is [22–24].

### Ethics statement

The registry used in this study was approved by the institutional review boards (IRBs) of SNU Hospital’s ethics committee (IRB no. 1605-150-768), SNU Bundang Hospital’s ethics committee (IRB no. B-1610-368-401), and SMG-SNU Boramae Medical center’s ethics committee (IRB no. 16-2016-123). The retrospective chart review study was performed with the approval of SNU Hospital’s ethics committee (IRB no. 1912-098-1089), and written consent was waived by the ethics committee of SNU Hospital. All methods were performed in accordance with the relevant guidelines and regulations.

## Results

### Baseline characteristics

**Fig 1:**
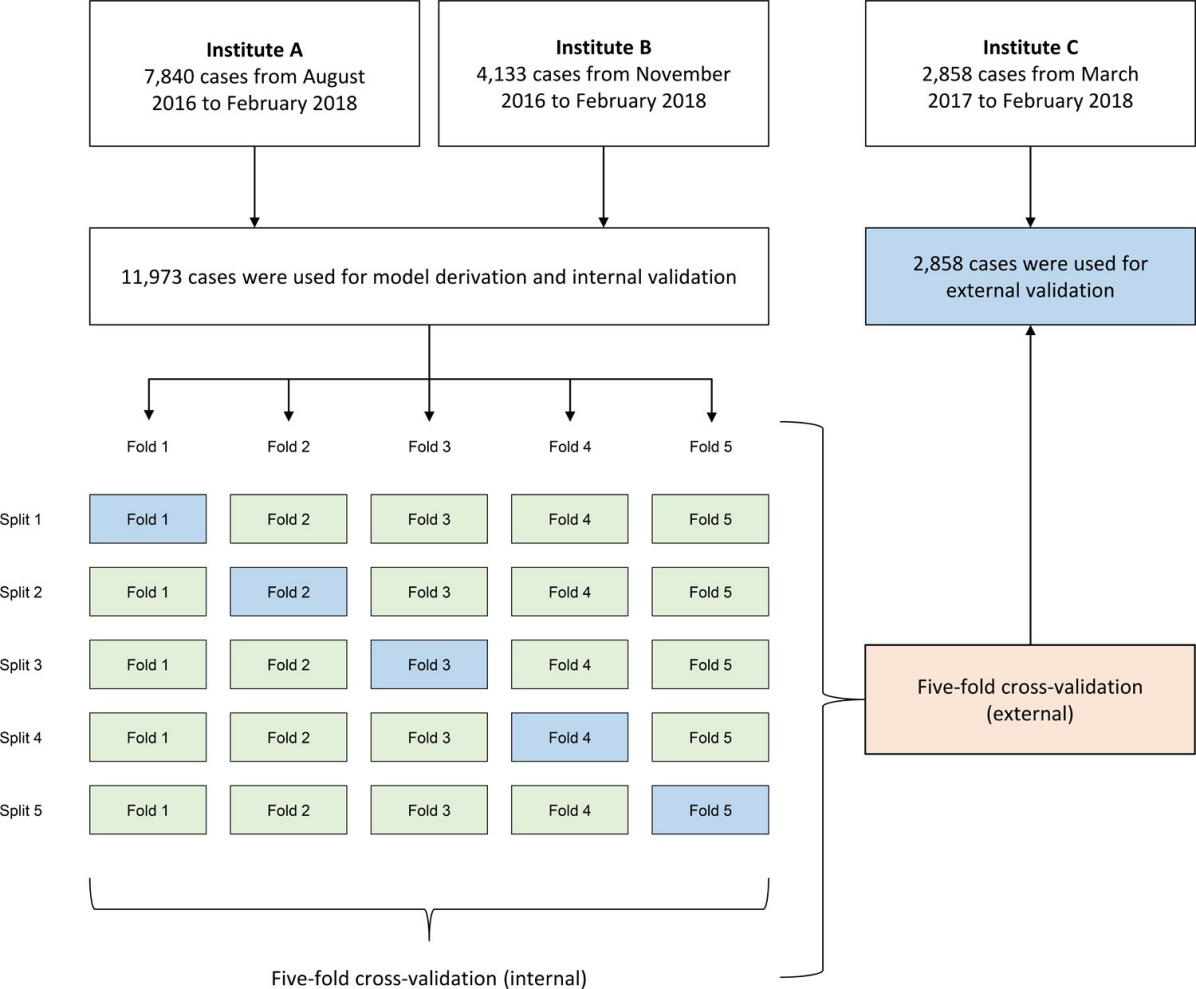
Flow chart of study subjects and the process of five-fold cross validation.

A total of 11,973 individuals were registered in the derivation cohort, the median (interquartile range [IQR]) age was 20 (11–37) months old, and 45.7% were female. The number of patients in the validation cohort was 2,858, the median (IQR) age was 21 (12–35) months old, and 45.9% were female. The 5-fold cross-validation process and the flow chart of each cohort are shown in Fig 1. The characteristics of each cohort, such as clinical findings and physical and laboratory examination results, are shown in Table 1.

**Table 1:** Baseline characteristics of datasets.

Variables				Derivation cohort (n = 11,973)	Validation cohort (n = 2,858)
Age, months				20.0 (11.0–37.0)	21.0 (12.0–35.0)
Female				5,467 (45.7)	1,312 (45.9)
Clinical findings	Fever duration, hours			24.0 (12.0–48.0)	24.0 (7.0–48.0)
	Immunizations administered as recommended schedule			9,822 (95.9)	1,258 (91.8)
	Attends day care center			3,995 (40.8)	900 (70.1)
Physical examination findings	Heart rate, beats/minute			150.0 (133.0–167.0)	140.0 (129.0–153.0)
	Respiratory rate, breaths/minute			30.0 (26.0–36.0)	22.0 (20.0–28.0)
	Body temperature, ℃			38.3 (37.7–39.0)	38.3 (37.7–39.0)
	Rash			703 (6.5)	119 (4.3)
Laboratory examination finding	Leukocyte, cells/mm^3^			7,365.0 (1,550.0–12,355.0)	10,350.0 (6,515.0–14,425.0)
	C-reactive protein, mg/dL			1.0 (0.3–3.0)	0.9 (0.3–2.3)
	Procalcitonin, μg/L			1.8 (1.1–3.7)	0.1 (0.1–0.1)
	pH			7.4 (7.4–7.4)	7.4 (7.4–7.4)
	Urinalysis	Bacteriuria		786 (14.0)	126 (14.3)
		Leukocyte esterase	Negative	4,429 (79.0)	652 (74.0)
			Trace	250 (4.5)	68 (7.7)
			1+	373 (6.7)	70 (7.9)
			2+	264 (4.7)	45 (5.1)
			3+	288 (5.1)	46 (5.2)
		Urine culture performed		2,756 (23.0)	764 (26.7)
	Blood culture performed			3,470 (29.0)	952 (33.3)
	Cerebrospinal fluid examination performed			208 (1.7)	2 (0.1)
Serious bacterial infection	Bacteremia			26 (5.6^a^)	0 (0.0^a^)
	Urinary tract infection			434 (93.1^a^)	93 (98.9^a^)
	Lobar pneumonia			4 (0.9^a^)	1 (1.1^a^)
	Bacterial CNS infection			1 (0.2^a^)	0 (0.0^a^)
	Septic arthritis			1 (0.2^a^)	0 (0.0^a^)

Continuous data are presented as median (interquartile range) and categorical data as number (%).

^a^Percentage of each item in all serious bacterial infection cases.

CNS, central nervous system.

### Main outcomes

**Fig 2:**
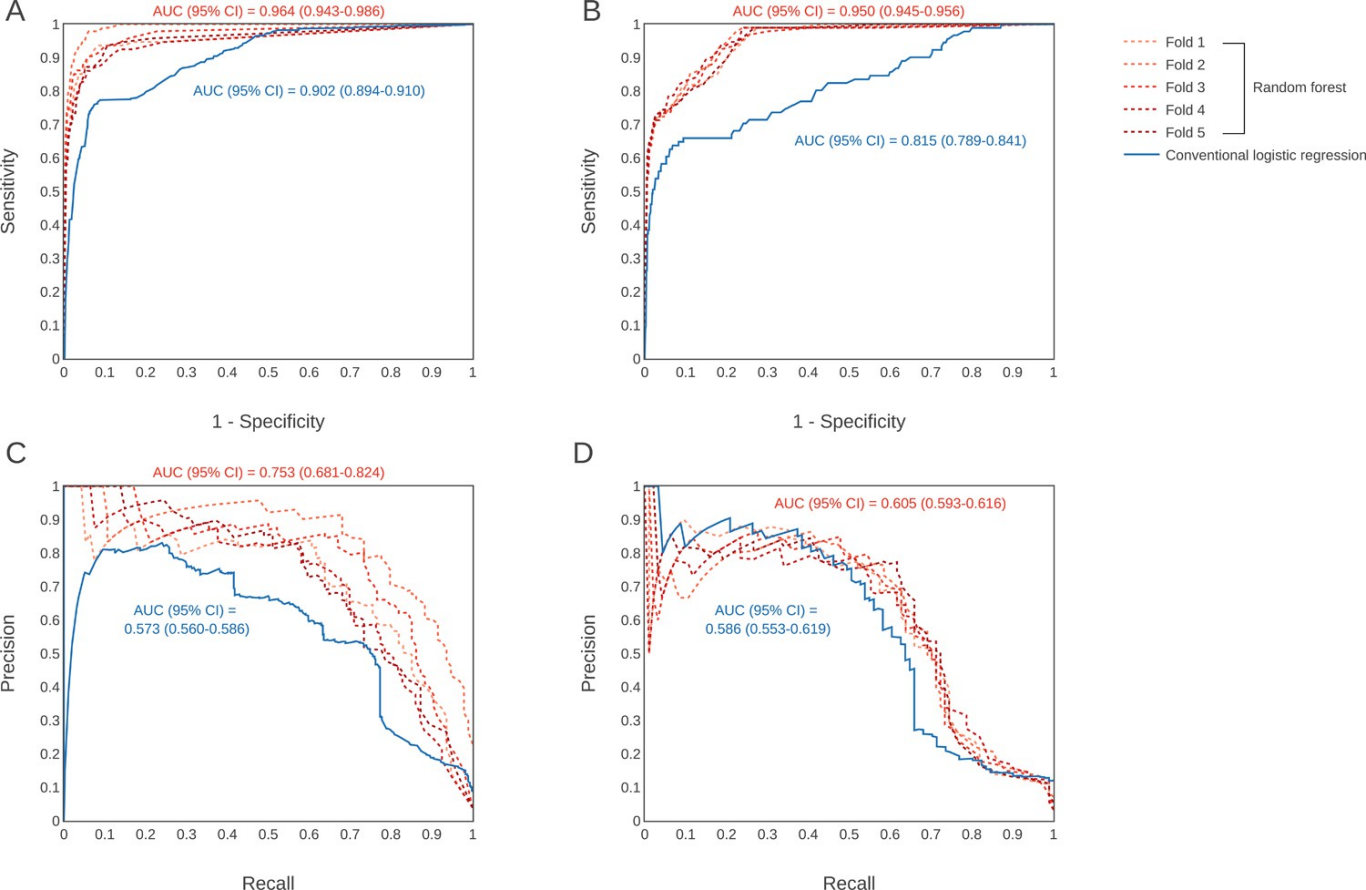
Internal and external validation of predictive models. The area under the receiver operating characteristics curves of the derivation cohort (A), the curves of the validation cohort (B), and the area under the precision-recall curves of the derivation cohort (C) and validation cohort (D) are shown. AUC = the area under the curve, CI = confidence interval.

The AUROC (95% confidence interval [CI]) in the validation cohort performed for external validation, the primary outcome of this study, was 0.950 (0.945–0.956) in the RF model and 0.815 (0.789–0.841) in the CLR model, which was higher in the RF model (Fig 2B). The AUPRC (95% CI) value was also high in the RF model at 0.605 (0.593–0.616) for the RF model and 0.586 (0.553–0.619) for the CLR model (Fig 2D). The AUROC in the derivation cohort was 0.964 (0.943–0.986) in the RF model and 0.902 (0.894–0.910) in the CLR model (Fig 2A). The AUPRC was 0.753 (0.681–0.824) and 0.573 (0.560–0.586), respectively (Fig 2C).

### Important factors for predicting SBI

**Fig 3:**
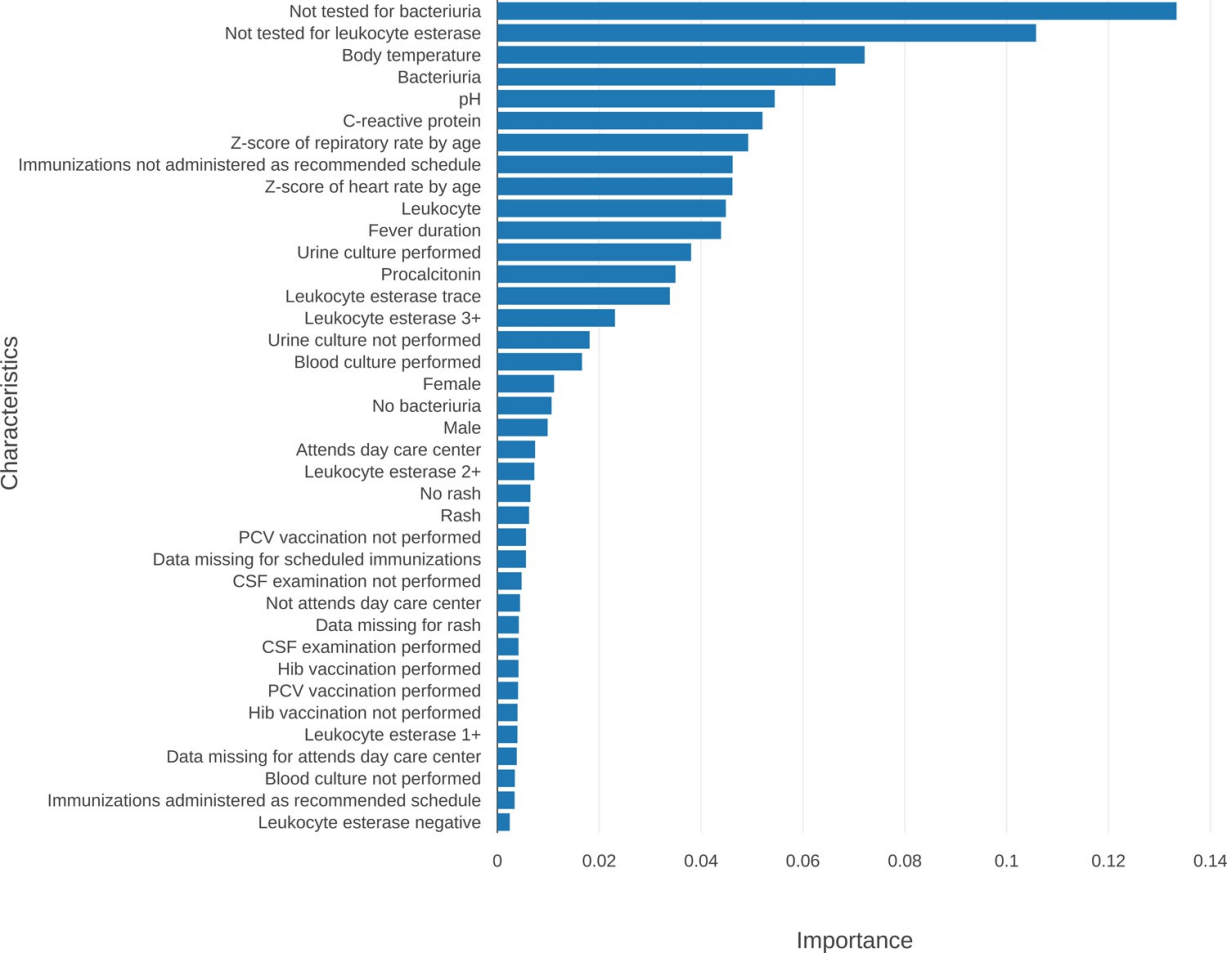
Feature importance of the RF model using the reduction in GINI impurity. Important factors for SBI prediction are listed in the order of importance, and the feature importance was obtained using the scikit-learn library [20]. CSF = cerebrospinal fluid.

In the feature importance of the RF model using the Gini impurity difference, bacteriuria and leukocyte esterase were not tested, and body temperature, bacteriuria, pH, and CRP were important features (Fig 3).

In the CLR model, bacteriuria, urine culture performed, and leukocyte esterase positivity were significant factors in multivariable analysis (Table 2).

**Table 2:** Conventional logistic regression analysis.

Variables		Univariable analysis			Multivariable analysis		
		OR	95% CI	*P*	OR	95% CI	*P*
Age, months		0.902	0.892–0.910	<0.001			
Sex	Female	Reference			Reference		
	Male	1.687	1.388–2.050	<0.001	4.268	0.893–20.388	0.069
Fever duration, hours		0.996	0.993–0.998	0.001			
Immunizations administered as recommended schedule^a^		0.547	0.354–0.845	0.007			
*Pneumococcal* Vaccination^a^		0.321	0.264–0.390	<0.001			
*Haemophilus influenzae* type b vaccination^a^		0.322	0.265–0.391	<0.001			
Attends day care center^a^		0.172	0.119–0.248	<0.001			
Rash^a^		0.282	0.145–0.548	<0.001			
Heart rate, beats/minute		1.016	1.012–1.020	<0.001			
Respiratory rate, breaths/minute		1.056	1.046–1.065	<0.001			
Body temperature, ℃		0.876	0.795–0.966	0.008	2.167	1.198–3.921	0.011
Leukocyte, cells/mm^3^		1.000	1.000–1.000	0.075			
C-reactive protein, mg/dL		1.089	1.068–1.110	<0.001			
pH		8.598	1.272–58.101	0.027			
Procalcitonin, μg/L		1.001	1.000–1.001	0.003			
Bacteriuria^a^		36.992	29.150–46.945	<0.001			
CSF examination performed^a^		5.478	3.777–7.945	<0.001			
Blood culture performed^a^		39.479	27.362–56.962	<0.001			
Urine culture performed^a^		152.482	85.804–270.977	<0.001	10.906	1.044–113.932	0.046
Leukocyte esterase	Negative	Reference			Reference		
	Trace	NA	NA	NA	NA	NA	NA
	1+	8.133	5.839–11.328	<0.001	12.455	2.372–65.393	0.003
	2+	24.402	17.834–33.387	<0.001	9.266	0.644–133.354	0.102
	3+	80.037	58.667–109.191	<0.001	102.649	15.377–685.242	<0.001

^a^As a categorical variable, ‘Yes’ was analyzed with ‘No’ as a reference for each item.

OR, odds ratio; CI, confidence interval; NA, not applicable; CSF, cerebrospinal fluid.

### Missing values in categorical variables

Among the categorical variables used in the analysis, missing values existed in ‘immunizations administered as recommended schedule’, ‘attends day care center’, ‘rash’, ‘bacteriuria’, and ‘leukocyte esterase’, and accounted for up to 69.2% (bacteriuria and leukocyte esterase items of the validation cohort) (Table 3). On the other hand, the case where the bacteriuria and leukocyte esterase tests were not performed (ie, missing) corresponded to the two most crucial factors in predicting SBI (Fig 3).

**Table 3:** Values of categorical variables used in the analysis.

Variables		Derivation cohort (n = 11,973)	Validation cohort (n = 2,858)
Sex	Female	5,467 (45.7)	1,312 (45.9)
	Male	6,506 (54.3)	1,546 (54.1)
Immunizations administered as recommended schedule	Yes	9,822 (82.0)	1,258 (44.0)
	No	422 (3.5)	112 (3.9)
	Missing	1,729 (14.4)	1,488 (52.1)
Attends day care center	Yes	3,995 (33.4)	900 (31.5)
	No	5,789 (48.4)	384 (13.4)
	Missing	2,189 (18.3)	1,574 (55.1)
*Pneumococcal* vaccination	Yes	10,059 (84.0)	2,858 (100.0)
	No	1,914 (16.0)	0 (0.0)
*Haemophilus influenzae* type b vaccination	Yes	10,054 (84.0)	2,858 (100.0)
	No	1,919 (16.0)	0 (0.0)
Rash	Yes	703 (5.9)	119 (4.2)
	No	10,151 (84.8)	2,660 (93.1)
	Missing	1,119 (9.3)	79 (2.8)
Bacteriuria	Yes	786 (6.6)	126 (4.4)
	No	4,818 (40.2)	755 (26.4)
	Missing	6,369 (53.2)	1,977 (69.2)
CSF examination performed	Yes	208 (1.7)	2 (0.1)
	No	11,765 (98.3)	2,856 (99.9)
Blood culture performed	Yes	3,470 (29.0)	952 (33.3)
	No	8,503 (71.0)	1,906 (66.7)
Urine culture performed	Yes	2,756 (23.0)	764 (26.7)
	No	9,217 (77.0)	2,094 (73.3)
Leukocyte esterase	Negative	4,430 (37.0)	652 (22.8)
	Trace	249 (2.1)	68 (2.4)
	1+	373 (3.1)	70 (2.4)
	2+	264 (2.2)	45 (1.6)
	3+	288 (2.4)	46 (1.6)
	Missing	6,369 (53.2)	1,977 (69.2)

Data are presented as number (%).

CSF, cerebrospinal fluid.

## Discussion

In this study, we developed a machine learning-driven RF model to predict SBI among febrile children under 6 years old in EDs and internally and externally validated the model. The predictive performance was good and seemed to be superior to that of the model derived by CLR in both the derivation and validation cohorts. To the best of our knowledge, this study is one of the first-generation trials to develop a clinical prediction model with a machine learning method to predict SBI in children [25, 26]. The implication of our study can be summarized in three parts: accuracy, applicability and validity.

In terms of accuracy, the results of our study showed excellent performance in both the derivation and validation cohorts. Our study also showed comparable performance to recently developed scoring systems that predict SBI in children. In a multicenter study by Dr. Kuppermann et al., the authors derived and validated a prediction rule to identify febrile infants 60 days and younger at low risk for SBIs using urinalysis, ANC, and PCT levels. They used the ‘recursive partitioning modeling’ method and showed the accuracy as follows; sensitivity of 97.7% (95% CI, 91.3–99.6), specificity of 60.0% (95% CI, 56.6–63.3), negative predictive value of 99.6% (95% CI, 98.4–99.9), and negative likelihood ratio of 0.04 (95% CI, 0.01–0.15) [8]. Unfortunately, the direct comparison for accuracy with our study was not possible because the performance of our study was presented with the AUROC and AUPRC. However, roughly, the ‘class’ of the accuracy of both studies seems to be ‘excellent’. Another recent study on the ‘refined Lab-score’ was reported by Dr. Leroy et al. In this multicenter cohort study of children with fever without a source, the authors used a ‘multilevel regression model’ with CRP, PCT, age and urinary dipstick analysis as independent variables. The accuracy of the model was indicated by an AUROC of 0.94 (95% CI = 0.93–0.96) [7], which is comparable with that in our study. With accuracy of the developed prediction rules, we also found differences in the target population. As shown before, our model was developed for the children under 6 years old. When comparing with ‘febrile infants rule (younger than 60 days)’ and ‘refined Lab-score (less than 3 years old)’, our model has an advantage for wider range of target population.

With regard to applicability, our methodology has a strong advantage for handling missing values. One of the significant aspects of our study is that missing values themselves were recognized as new variables and used for learning. In the existing conventional method, missing values are excluded from model training or imputed. Consequently, they are considered a handicap in terms of prediction model development. However, in this study, the clinical significance of the absence of a specific variable was highlighted, and the missing value itself was used to develop a predictive model that played a role as a variable with clinical significance. In fact, in the process of developing the ‘clinical prediction rule’ for predicting SBI in infants under 60 days mentioned above, 1,334 (41%) out of 3,230 eligible participants were excluded from analysis due to missing values [8]. In the ‘Lab score’ study, 1,619 (50%) of 3,244 eligible individuals were also excluded due to missing values [27]. The predictive powers of these studies were excellent; however, if a predictive model cannot be applied to approximately 40%–50% of eligible patients, its significance in terms of actual clinical application is bound to be very limited. As we showed in our results, the RF model could be applicable to more patient records.

The third part is the validity of the model when considering the parameters of the adopted variables. Although the machine learning algorithm may not seem easy to understand, there is the mutual similarity of important features between the RF model and multivariable LR. The presence or absence of bacteriuria, whether urine culture was performed, and the grade of leukocyte esterase were also significant factors in multivariable LR, and most of them were highly ranked for the feature importance of the RF model. Interestingly, whether urine culture was performed was recognized as a significant factor in both models. If the model was developed only based on the urine culture results, however, if urine culture was not performed, the value would have been missing and may have undergone a process such as imputation. However, in this study, the missing value, itself, played a significant role with statistical power and clinical significance. This similarity of variables might support the validity of our modeling method.

Finally, we compared our model with the CLR method because CLR was the most commonly (so, it is conventional) used way to develop a predictive model before the machine learning era. Although the CLR model showed relatively lower performance than the RF model in both internal and external validation, the values of the AUROC of 0.815–0.902 are not low. There could be multiple reasons why CLR in this study also showed a relatively high AUROC. First, we used somewhat formalized data from the registry type dataset. Second, majority of SBIs was UTI, and the prediction seemed to be rather straightforward. For this reason, the feature extraction process in this study was relatively simple. If it was image data or a predictive model was developed based on more complex unstructured data, we think it would have been possible to develop a better performing model using feature extraction techniques such as ‘orthogonal moments’ [28–30].

This study had several limitations. First, UTI accounted for majority of SBIs in this study because of the reduced incidences of respiratory and invasive bacterial infections in Korea, as a result of the high immunization rates of the *H*. *influenzae* type b vaccine and PCV, which are included in the national immunization program [31]. Second, the data used for learning in this RF model were generally formalized information recorded in the registry. If the model was developed using methods such as natural language processing for unstructured data, the difference between the machine learning model and the CLR model could have been further highlighted. Third, the great majority of the enrolled cases were Korean children living in relatively homogenous lifestyle, which means that this population does not represent ethnic, racial or cultural diversity. The external validation of this prediction model in more diverse pediatric population group is warranted.

## Conclusions

The RF model of this study, which was developed to predict SBI even with missing values by including missing values in the model development, showed excellent performance for predicting SBI among febrile children in the ED. Our methodology had a strong advantage for handling missing values, and the missing value, itself, played a significant role with statistical power and clinical significance. A better performance was observed than the CLR model. Further studies including more patients, wider areas, and more diverse bacterial infections are warranted.

## Supporting information

S1 Data (XLSX)
